# *Aedes vittatus* in Haiti: continued expansion across the Caribbean and implications for the Americas

**DOI:** 10.1590/0074-02760260238

**Published:** 2026-10-19

**Authors:** Daphenide St-Louis, Philippe Boussès, Loïc Talignani, Chrystelle Lasica, Evens Emmanuel, Frédéric Simard, David Roiz

**Affiliations:** 1 Université de Montpellier, Centre National de la Recherche Scientifique, Institut de Recherche pour le Développement, Maladies infectieuses et vecteurs: écologie, génétique, évolution et contrôle, Montpellier, France; 2 Quisqueya University, Climate Change Research Team, Port-au-Prince, Haiti; 3 Consejo Superior de Investigaciones Científicas-Universidad de Castilla-La Mancha-Junta de Comunidades de Castilla-La Mancha, Instituto de Investigación en Recursos Cinegéticos, Sanidad y Biotecnología, Ciudad Real, Spain

**Keywords:** arboviruses, invasive mosquitoes, Haiti, chikungunya, dengue, Aedes, vittatus

## Abstract

**BACKGROUND:**

*Aedes vittatus*, an emerging invasive species and arboviral vector with high ecological plasticity and dispersal capacity, is expanding in the Caribbean, being detected in the Dominican Republic, Cuba, and Jamaica and recently in Yucatán, Mexico.

**OBJECTIVES:**

This study aimed to provide evidence of the establishment of *Ae. vittatus* in Haiti.

**METHODS:**

Larval surveys were conducted in 18 localities within 12 municipalities in the North and Northeast departments of Haiti. Specimens were identified morphologically and confirmed by COI-based DNA barcoding.

**FINDINGS:**

We report, for the first time, the detection of the invasive vector *Ae. (Fredwardsius) vittatus* in Haiti, confirmed morphologically and molecularly, with the species detected in half of the municipalities sampled. Phylogenetic analyses placed Haitian specimens within a Caribbean/American group, closely related to sequences from Mexico. Additional mosquito species were identified, including first records of *Culex garciai* and *Culex interrogator* in Haiti.

**MAIN CONCLUSIONS:**

Given its potential role as a vector of chikungunya, dengue, Zika and yellow fever, continued entomological surveillance and early detection in the Caribbean and the Americas are needed.

Invasive vector mosquito species, such as *Aedes aegypti* and *Aedes albopictus*, have spread globally through human activities, increasing the public health and economic burden of arboviral diseases such as dengue, chikungunya and Zika.^(^ ^1^ ^,^ ^2^ ^)^ *Aedes vittatus*, an emerging invasive species and arboviral vector with high ecological plasticity and dispersal capacity, was first detected in the Caribbean in 2019 in the Dominican Republic and Cuba, followed by Jamaica in 2023 and Mexico in 2024-2025.^(^ ^3^ ^,^ ^4^ ^,^ ^5^ ^)^ These records indicate rapid expansion across the Caribbean basin and into the Americas. Despite demonstrated vector competence, the species remains poorly studied.^(^ ^4^ ^,^ ^5^ ^)^ Haiti, characterised by high population density, environmental change, and intense human mobility, provides favourable conditions for mosquito invasions; however, *Ae. vittatus* had not previously been reported there. We report the first detection and evidence of establishment of *Ae. vittatus* in Haiti.

## MATERIALS AND METHODS

*Study area* - The survey was conducted in the North and Northeast departments of Haiti, restricted to the extensive, low-elevation coastal zones of both regions. Following the 2010 earthquake that struck the Port-au-Prince area, the North department experienced the arrival of more than 2.3 million people, resulting in rapid and largely unplanned urban expansion, increasing the suitable area for urban mosquitoes.^(^ ^6^ ^)^ This department is therefore densely populated (500 inhabitants/km²), in contrast to the Northeast, where population density is approximately 240 inhabitants/km². The climate varies from tropical monsoon (North Department) to tropical savanna (Northeast Department), with mean annual temperatures of 24 to 28ºC and seasonal rainfall (April to November), averaging between 1,000 and 1,500 mm per year, with a decreasing gradient from the coast toward the interior.

The study area, located mainly within the coastal plains surrounding the departmental capital, Cap-Haïtien, extends from Limbé (19º42’N, 72º24’W) in the west to Ouanaminthe (19º29’N, 71º46’W)**,** located on the Dominican border, 75 km east of Limbé, following the coastal fringe and reaching up to 20 km inland. It is characterised by moderately low relief, with most of the coastline and its surroundings lying at elevations generally below 100 m in urban and peri-urban zones.

*Mosquito sampling* - Between October 7 and October 31, 2025, during the latter part of the rainy season, surveys were conducted in urban, peri-urban and rural areas across both departments. A total of 23 larval habitats were collected in 18 localities within 12 communes (seven in the North and five in the Northeast) (Fig. 1). Sampling time did not exceed 3 h per commune. Mosquito larvae were collected from peridomestic and public spaces. For each sample site, some collected larvae were mounted on slides, while others were reared to the adult stage, and both types of specimens were deposited in the ARIM-IRD collection.^(^ ^7^ ^)^ Geographical coordinates and data were uploaded to the GBIF platform.

**Fig. 1:**
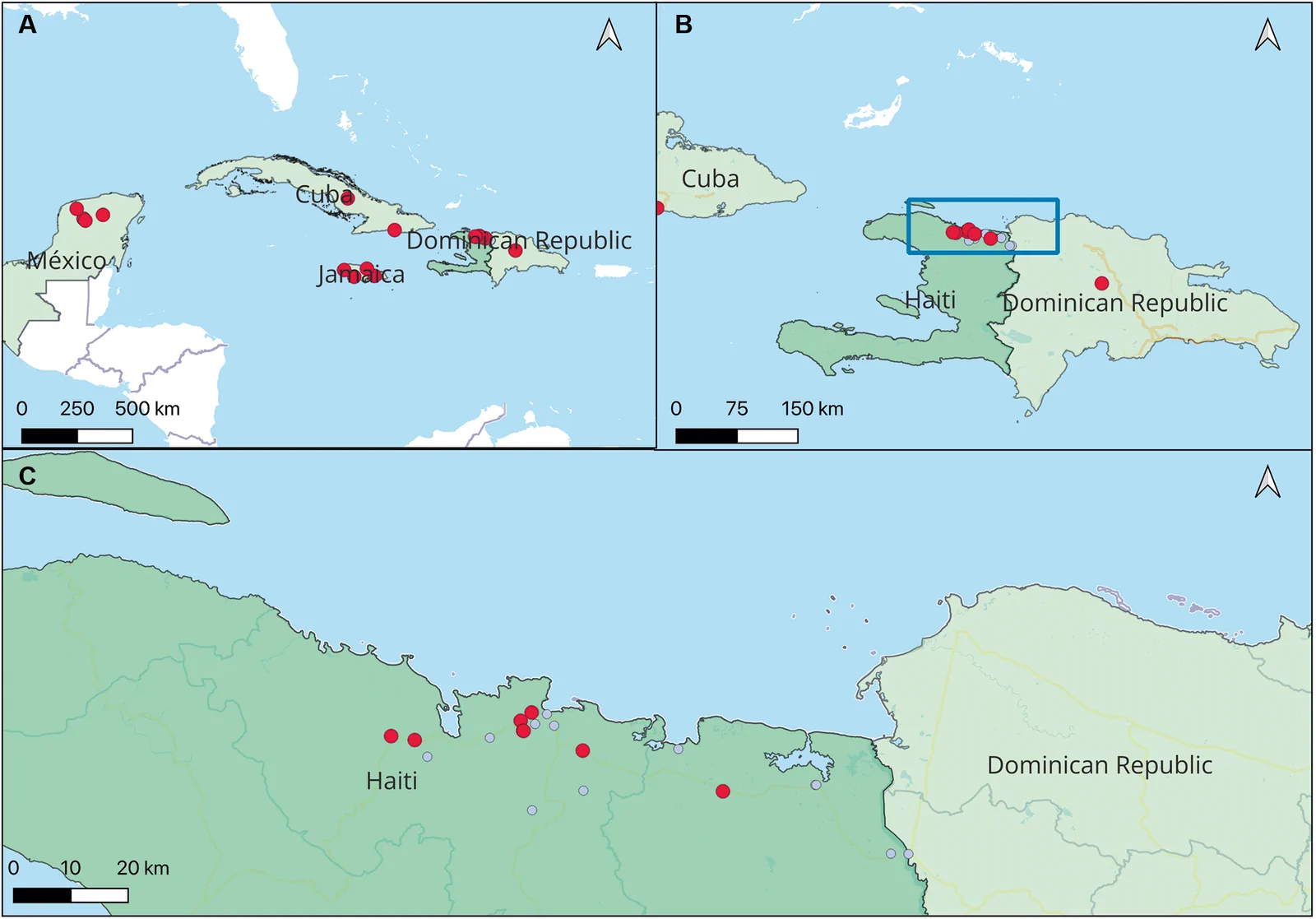
location of the sampling sites. (A) Situation map of Haiti within Caribbean countries with presence (green area) and location of capture points (red dots) of *Aedes vittatus* in neighbouring countries; (B) Delimitation of study area in Haiti (blue rectangle) and location capture (red dots) of *Ae. vittatus* in Haiti and the Dominican Republic; (C) Location of the breeding site of *Ae. vittatus* in the study area (red dots) and other collection sites negative to *Ae. vittatus* (grey dots) (Table).

*Ethics statement* - This study was conducted with authorisation from the Haitian Ministry of Environment, and the importation of mosquitoes from Haiti to France was approved by the Departmental Directorate for Population Protection (DDPP).

*Morphological and molecular identification* - The invasive species *Ae. vittatus* was identified using European,^(^ ^8^ ^)^ African^(^ ^9^ ^)^ and Sahelian^(^ ^10^ ^)^ taxonomic keys, and confirmed with the species redescription of Huang.^(^ ^11^ ^)^ Five samples from the localities Quartier Morin (two adults: QUA003-Haiti06 and 07) and Lory, Milot (two adults: MIL001-Haiti08 and 09 and one larvae: MIL004LAV-10) were used for molecular identification. Mosquito DNA was extracted with the Qiagen Blood&tissue kit (Qiagen, Valencia, USA). Mosquitoes were individually homogenised with three metal beads in a Qiagen TissueLyserII at 30 Hz for 3 min. The remainder of the protocol followed the manufacturer’s instructions. Polymerase chain reaction (PCR) was performed in duplicate for each sample to validate the species identification through a universal invertebrate-specific primer pair LCO1490 (5′-GGT CAA CAA ATC ATA AAG ATA TTG G-3′) and HCO2198 (5′-TAA ACT TCA GGG TGA CCA AAA AAT CA-3′) to amplify a 658-bp fragment of the mitochondrial gene cytochrome c oxidase subunit I barcoding region (COI).^(^ ^12^ ^)^ PCR reactions were performed in a total volume of 35 µL, containing 17.5 µL Invitrogen TM PlatinumTM Hot Start PCR 2X Master Mix, 0.35 µL of each primer, 9.8 µL of biology grade water, and 7 µL of DNA. PCR amplification products were sent to Macrogen (Macrogen Europe, Amsterdam, the Netherlands) for Sanger sequencing. A Basic Local Alignment Search Tool (BLAST) analysis was conducted in the National Centre for Biotechnology Information (NCBI) GenBank database to explore species identification based on the amplified and sequenced molecular product.^(^ ^13^ ^)^

*Sequence assembly, alignment and phylogenetic analyses* - Forward and reverse reads of four samples, as one of the five samples (MIL004LAV-10) did not amplify, were assembled and edited to generate consensus sequences using CodonCode Aligner v12.0.4 (https://www.codoncode.com/aligner). Sequence alignments were performed with MAFFT v7 via the online server (https://mafft.cbrc.jp/alignment/server/) using default parameters.^(^ ^14^ ^)^ The resulting alignments were visually inspected and manually edited in AliView v1.28.^(^ ^15^ ^)^ The best-fit nucleotide substitution model was determined using jModelTest2 through the CIPRES Science Gateway (https://www.phylo.org/) under default settings.^(^ ^16^ ^)^ Mitochondrial sequences, including COI marker sequences of *Aedes* spp., were retrieved from GenBank (accession numbers provided in the phylogenetic tree). The dataset comprised sequences from Africa, Asia, Europe, the Caribbean, and a recently generated sequence from Mexico.

Phylogenetic relationships among *Ae. vittatus* sequences were inferred using BEAST v1.10.5, applying a General Time Reversible substitution model+r returned by jModelTest2, a strict molecular clock, and a coalescent constant size tree prior. *Psorophora ferox* was designated as the outgroup.^(^ ^17^ ^)^ Markov chain Monte Carlo (MCMC) analyses were run for 10 million generations, with trees sampled every 100 generations. Effective sample sizes (ESS) were assessed using Tracer v 1.7.2,^(^ ^18^ ^)^ with values > 200 considered adequate. Convergence and stationarity were assessed by examining parameter traces, and the first 25% of samples were discarded as burn-in prior to summarising posterior probabilities. The final phylogenetic tree was visualised and edited using FigTree v1.4.4.^(^ ^19^ ^)^

*Network analysis* - Analyses at the intraspecific level were performed on datasets that encompassed only *Ae. vittatus* sequences. The genetic differentiation between specimens belonging to the major phylogroups inferred by the phylogenetic analyses of the combined dataset was assessed for each COI gene using DnaSP v.6.12.03.^(^ ^20^ ^)^ Haplotype networks were constructed using the median-joining method,^(^ ^21^ ^)^ which has the ability to deal with missing data as well as to infer ancestral haplotypes. This method also performs well against or outperforms other network approaches.^(^ ^22^ ^)^ It was implemented using the software PopART v1.7,^(^ ^23^ ^)^ with epsilon value set to 0 in order to minimise alternative median networks. The resulting networks were visualised and edited in Inkscape v1.2.2 (https://inkscape.org/).

## RESULTS

During the sampling period, *Ae. vittatus* was detected in eight of the 23 sites (34.7%) across six municipalities in northern Haiti: Cap-Haïtien, Quartier Morin, Milot, Limbé, Trou du Nord, and Terrier Rouge (Table). The species was found alone in three of these sites and co-occurred with *Ae. albopictus* (three sites) and *Ae. aegypti* (two sites). Breeding habitats included diverse peridomestic and public environments across urban, peri-urban, and rural settings, such as water storage containers, groundwater accumulations, poultry waterers, wastewater channels, puddles, and tree root cavities. These habitats were generally devoid of vegetation and varied in water volume and organic content. Eight additional mosquito species were recorded, including *Ae. aegypti*, *Ae. albopictus*, *Culex quinquefasciatus*, *Culex garciai*, *Culex interrogator*, *Culex nigripalpus*, *Psorophora confinnis* and *Psorophora pygmaea*. Note that *Cx. garciai* and *Cx. interrogator* constitute new country records for Haiti.

**TABLE:** Samples and mosquito species collected in the North and Northeast departments of Haiti

Locality	Municipality	Sample ID	Latitude	Longitude	Habitat	Date of collection	Mosquito species
Rue de l’Union	Cap-Haïtien	CAP012	19.72523	-72.21062	stagnant water around habitation	09/10/2025	*Culex garciai*
Madeline	Cap-Haïtien	CAP018	19.72299	-72.18501	water domestic container	13/10/2025	*Culex quinquefasciatus*
Near airport Cap-Haïtien	Cap-Haïtien	CAP019	19.73843	-72.19466	stagnant water	14/10/2025	*Aedes aegypti Culex interrogator*
Babiole	Cap-Haïtien	CAP021	19.72956	-72.22979	200 L plastic drum	17/10/2025	*Aedes albopictus* ***Aedes vittatus***
Bois de lance	Limonade	LIM001	19.63604	-72.14587	stagnant water on the road	10/10/2025	*Culex garciai*
Carrefour Plaine du Nord	Plaine du Nord	PLN004	19.70675	-72.27112	stagnant water at home	13/10/2025	*Culex quinquefasciatus*
Cité Deloma	Terrier Rouge	TER001	19.63511	-71.95896	water plastic container	22/10/2025	*Aedes aegypti* ***Aedes vittatus***
Cité Deloma	Terrier Rouge	TER002	19.63452	-71.96019	tyre	14/10/2025	*Aedes aegypti Culex nigripalpus*
Dignitaire 3	Milot	MIL003	19.60977	-72.21449	garbage dump under a bridge	16/10/2025	*Aedes albopictus*
Lory	Milot	MIL004	19.7162	-72.22649	stagnant water in front of a house	24/10/2025	*Aedes albopictus* ***Aedes vittatus***
Lory	Milot	MIL008	19.71596	-72.22594	concrete poultry waterers	24/10/2025	*Aedes aegypti* ***Aedes vittatus***
Dupathy	Acul du Nord	ACN004	19.68142	-72.35479	artificial container	08/10/2025	*Aedes aegypti*
Fort-Liberté	Fort-Liberté	FOR001	19.64374	-71.83533	ox hoofprints	31/10/2025	*Psorophora confinnis*
Fort-Liberté	Fort-Liberté	FOR002	19.64374	-71.83391	wastewater canal	31/10/2025	*Culex quinquefasciatus*
Garcin	Trou du Nord	TDN002	19.74069	-72.21515	wastewater canal	07/10/2025	*Culex quinquefasciatus*
Garcin	Trou du Nord	TDN002	19.74069	-72.21515	wastewater canal 2	07/10/2025	***Aedes vittatus***
Marché Ouanaminthe	Ouanaminthe	DAJ001	19.55118	-71.71049	plastic plate	30/10/2025	*Aedes albopictus Aedes aegypti*
Ouanaminthe	Ouanaminthe	OUA001	19.55166	-71.73403	tyre in the peridomestic space	30/10/2025	*Culex quinquefasciatus Aedes aegypti*
Pont Caracol	Caracol	CAC004	19.69169	-72.01874	wastewater canal	21/10/2025	*Culex garciai*
Quartier Morin	Quartier Morin	QUA003	19.68958	-72.14669	rut on the road	22/10/2025	***Aedes vittatus***
Château neuf	Limbé	LIB001	19.70381	-72.37166	tree roots	14/10/2025	*Aedes albopictus* ***Aedes vittatus***
Simonète	Limbé	LIB002	19.70906	-72.40326	Stagnant water on road	14/10/2025	***Aedes vittatus***
Simonète	Limbé	LIB003	19.70906	-72.40326	Stagnant water on road	14/10/2025	*Psorophora pygmaea*

*Morphological identification* - Both larval stages and adults obtained from breeding were examined, and diagnostic characters for the principal life stages for *Ae. vittatus* are summarised as follows (Fig. 2).

**Fig. 2:**
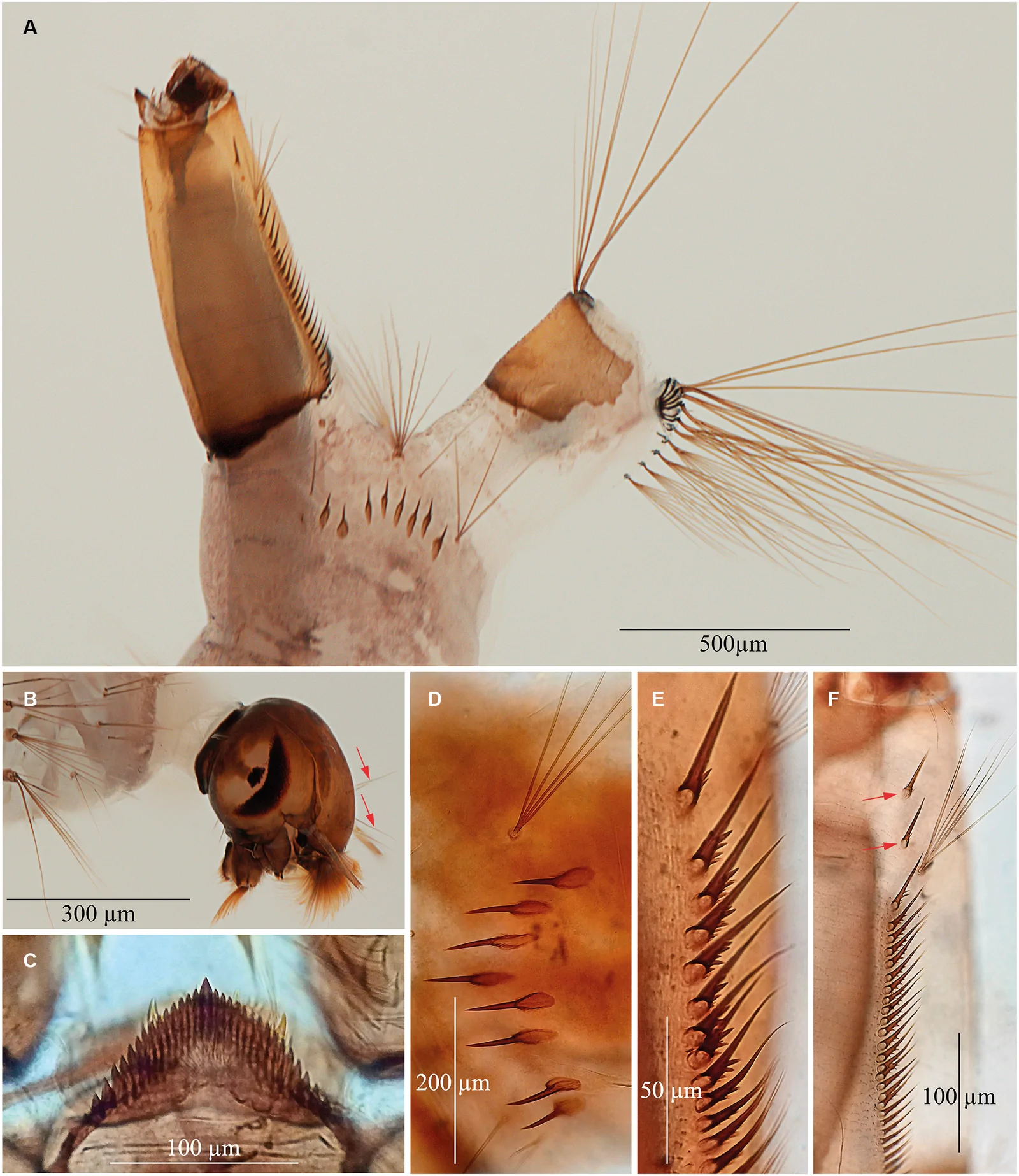
*Aedes vittatus*: morphological characteristics of the larval stage. (A) Detail of abdominal segments VIII-X (the long anal papillae are missing); (B) head showing single setae 5-C and 6-C (arrows); (C) mentum; (D) comb VIII with eight spines; (E) detail of the pecten teeth with two or three denticles; (F) atypical pecten with the rare presence of two teeth (instead of one, arrows) inserted distal to the 1-S setae.

*Female* - Head with a dark proboscis with a median scattering of pale yellowish scales. Thorax: scutum with acrostichal setae; three pairs of distinct, small, white spots of narrow scales on the anterior two-thirds of the scutum; scutellum with broad white scales on all three lobes; lower mesepimeral setae present. Legs: tibia with a median white ring, tarsomeres III-1-4 with large basal white bands, tarsomere III-5 entirely white. Abdomen: tergite I with a large median white spot.

*Male genitalia* - Gonocoxite elongated, without basal and apical lobes. The gonostyle has a very characteristic shape, strongly enlarged and globose at its tip, with a long, strongly curved apical spine located at the base of the globose part. The claspette is large, with a narrow base, and distinctly swollen from its middle.

*Larva (Fig. 2)* - Head: setae 5-C single or sometimes double, 6-C single. Abdomen: setae 6-I-IV usually with two long branches; pecten with 19-25 very closely spaced spines, with 3-4 basal denticles; only the most distal spine (rarely two) more widely spaced and implanted beyond seta 1-S; comb of eight (6-10) scales in a single irregular row; saddle incomplete without marginal spicules; 1-X single, 2-X with 5 branches; four precratal setae; anal papillae long, about four times as long as saddle, slender and tapered.

*Molecular identification* - Morphological identification was confirmed with COI-based DNA barcoding. The *Ae. vittatus* specimens (Haiti_06, Haiti_07, Haiti_08, Haiti_09) clustered with Caribbean and American haplotypes (Cuba, Jamaica, the Dominican Republic, and Mexico).^(^ ^3^ ^,^ ^5^ ^(24-27)^ In the haplotype network, they share closely related haplotypes separated by only a small number of mutations, suggesting a high level of genetic similarity within this group, particularly with Mexican populations^(^ ^5^ ^)^ (Fig. 3). This Caribbean/American cluster appeared distinct from African haplotypes and from most Asian haplotypes, which form separate groups within the network. The observed pattern is consistent with the Caribbean/American lineage being genetically distinct from African and most Asian haplotypes, although the limited number of specimens and the use of a single mitochondrial marker preclude firm conclusions about the geographic origin or pathways of introduction. The COI-based DNA barcoding confirmed the identification of *Ps. pygmaea*, *Cx. quinquefasciatus*, *Ae. aegypti*, *Ae. albopictus* and *Cx. interrogator*, with the latter showing high similarity to GenBank reference sequences (99.67% identity; MT999299.1). For *Cx. garciai*, no reference sequences are currently available in GenBank; therefore, we generated the first COI sequences for this species in this study. Sequences of *Ae. vittatus* were submitted to GenBank under accession numbers PZ344144, PZ344145, PZ344146 and PZ344147. Sequences of *Ae. aegypti*, *Ae. albopictus*, *Cx. garciai*, *Cx. interrogator*, *Cx. quinquefasciatus* and *Ps. pygmaea* have been deposited under accession numbers PZ878903, PZ878904, PZ878905, PZ878906, PZ878907, PZ878908, PZ878909, PZ878910, PZ878911.

**Fig. 3:**
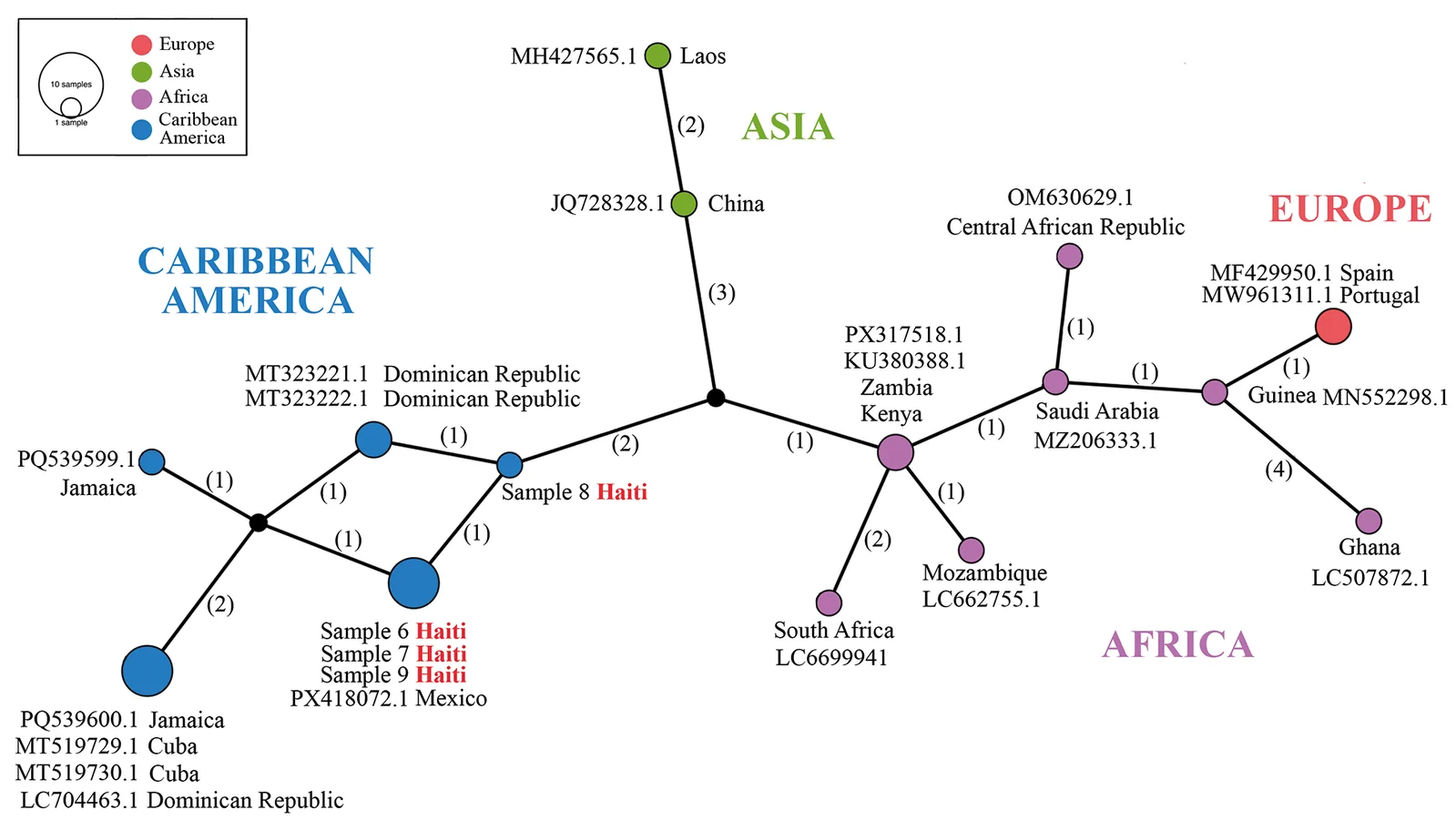
haplotype network and phylogenetic tree resulting from the analysis of the cytochrome c oxidase subunit I barcoding region (COI) dataset. The haplotype network reconstruction considers missing data and gap. Values between parentheses on nodes indicate the inferred number of mutation steps between haplotypes or ancestral haplotypes (symbolised by a black node). The phylogenetic tree corresponds to the results of a Bayesian inference analysis (see text for details).

## DISCUSSION

Species identification was confirmed morphologically and by COI-based DNA barcoding. Haitian *Ae. vittatus* specimens clustered with Caribbean and American haplotypes (Cuba, Jamaica, Dominican Republic, and Mexico) and were clearly distinct from African and most Asian lineages (Fig. 3). This pattern is consistent with a shared regional genetic background within the Caribbean/American group, although more molecular markers, more populations and specimens are needed to confirm this hypothesis. Detection of *Ae. vittatus* in one-third of the sampled habitats and half of the surveyed communes, despite limited sampling, indicates that the species is present at multiple localities in northern Haiti. However, given the limited sampling effort, further surveys are needed to assess its wider distribution and establish the extent of its establishment.

Its absence from earlier surveys in 2013,^(^ ^6^ ^)^ combined with its detection in the Dominican Republic in 2019,^(^ ^3^ ^)^ is consistent with a relatively recent arrival on Hispaniola. However, the available data do not allow us to determine the timing or pathway of introduction. Passive transport via water holding containers and other goods may have contributed to its spread, but the relative importance of each introduction pathway remains unknown. Passive transport via water-holding goods is a well-documented pathway for mosquito invasions, and the species’ desiccation-resistant eggs further enhance its dispersal potential. Genetic similarity with recently detected Mexican populations^(^ ^5^ ^)^ suggests strong regional connectivity, although the direction of spread remains unclear. Additional sampling across Haiti and the use of nuclear and/or genomic markers will be necessary to clarify the distribution, population structure and introduction pathways of the species.

Current socio-environmental conditions in Haiti, including population displacement, informal settlements, inadequate water infrastructure, and unmanaged waste, favour the proliferation of container-breeding mosquitoes and may provide favourable conditions for the establishment of *Ae. vittatus*. The species exhibits high ecological plasticity, colonising a wide range of artificial and natural habitats. Its tolerance to desiccation, salinity, and high temperatures further enhances its invasion potential.^(^ ^28^ ^)^ Co-occurrence with *Ae. aegypti* and *Ae. albopictus* raises questions about interspecific interactions and future ecological dynamics in urban environments. Notably, we also reported *Cx. garciai* and *Cx. interrogator* from Haiti for the first time. Morphological identification of *Cx. interrogator* was supported by molecular results, showing high sequence identity but lower query coverage, likely reflecting partial sequences or trimming.

From a public health perspective, *Ae. vittatus* is competent for multiple arboviruses, including dengue, chikungunya, Zika, and yellow fever.^(^ ^29^ ^,^ ^30^ ^)^ Its establishment adds complexity to Haiti’s epidemiologic context, which already includes endemic dengue, recurrent chikungunya outbreaks, and ongoing malaria and filariasis transmission. Notably, ecological niche models suggest that *Ae. vittatus* could find year-round suitable conditions across coastal regions of the Gulf of Mexico and extend into the southern Atlantic states of the United States, as well as large areas of Mexico and Central America.^(^ ^31^ ^)^ This potential geographic suitability highlights the possibility of further spread into North America, Central and South America and underscores the need for strengthened surveillance and preparedness beyond the Caribbean.

Our findings provide the first confirmed record of *Ae. vittatus* in Haiti and demonstrate its presence in multiple locations. Further surveillance is needed to determine its distribution, extent of establishment, ecological interactions, and potential contribution to arbovirus transmission in the region and beyond.

## Data Availability

Sequences of *Aedes vittatus* have been deposited in GenBank under accession numbers PZ344144, PZ344145, PZ344146 and PZ344147. Sequences of *Aedes aegypti*, *Aedes albopictus*, *Culex garciai*, *Culex interrogator*, *Culex quinquefasciatus* and *Psorophora pygmaea* have been deposited under accession numbers PZ878903, PZ878904, PZ878905, PZ878906, PZ878907, PZ878908, PZ878909, PZ878910, PZ87891.
