# Supplementary material for: Aedes vittatus in Haiti: continued expansion across the Caribbean and implications for the Americas
Source: Mem Inst Oswaldo Cruz. 2026 Oct 19;121:e260238. doi: 10.1590/0074-02760260238 (PMC13682729; doi:10.1590/0074-02760260238)
Supplement: Peer review history

# Reviewer report: FIRST REVIEW ROUND - REVIEWERS’ COMMENTS

## REVIEWER #1

The abstract adequately summarizes the study and highlights the first detection of Aedes vittatus in Haiti. This finding fills an important geographical gap and has clear relevance for mosquito surveillance in the Caribbean and the Americas. The manuscript should avoid strong conclusions, as these interpretations are based on limited sampling and a single mitochondrial marker. Inconsistencies in dates and sites / localities should also be corrected. References are generally relevant and updated, but their formatting should be standardized.

## AUTHORS’ RESPONSE TO THE REVIEWERS

Dear Prof. Ricardo Lourenço-de-Oliveira and Maria Clara Carvalho:

We are pleased to submit the letter of response to the referees for the manuscript entitled “Aedes vittatus in Haiti: continued expansion across the Caribbean and implications for the Americas” for consideration in Memórias do Instituto Oswaldo Cruz, on behalf of my co-authors. Here are our point-by-point responses. The comments are in bold and our responses are in regular text in blue. We have added the modifications to a track changes version of the manuscript in red.

Reviewer comments:

The abstract adequately summarizes the study and highlights the first detection of Aedes vittatus in Haiti. This finding fills an important geographical gap and has clear relevance for mosquito surveillance in the Caribbean and the Americas.

We sincerely thank you for your positive and constructive and positive comments. We are pleased that you find the study relevant and that the detection of Aedes vittatus in Haiti fills an important geographical gap for mosquito surveillance in the Caribbean and the Americas.

The manuscript should avoid strong conclusions, as these interpretations are based on limited sampling and a single mitochondrial marker.

We agree and we have moderated the strength of our conclusions throughout the manuscript, particularly in the Abstract and Discussion, to better reflect the limitations of our study, including the relatively limited sampling effort and the use of a single mitochondrial marker (COI). We now explicitly acknowledge these limitations and clarify that our interpretations regarding establishment, regional expansion and spread should be considered with caution.

In the abstract, we changed for: “We report, for the first time, the detection of the invasive vector Aedes (Fredwardsius) vittatus in Haiti, confirmed morphologically and molecularly, with the species detected in half of the municipalities sampled. Phylogenetic analyses placed Haitian specimens within a Caribbean/American group, closely related to sequences from Mexico.” (lines 31-34). Also in the main conclusion of the abstract: “Given its potential role as a vector of chikungunya, dengue, Zika and yellow fever, continued entomological surveillance and early detection in the Caribbean and the Americas are needed.” (lines 35-36).

In the results, we substitute the sentence: “The results support a common Asian-rather than African or European origin with regional spread in the Caribbean.”, with “The observed pattern is consistent with the Caribbean/American lineage being genetically distinct from African and most Asian haplotypes, although the limited number of specimens and the use of a single mitochondrial marker preclude firm conclusions about the geographic origin or pathways of introduction.” (lines 172-176).

In the conclusion, we substitute: “This pattern supports a shared regional origin and spread within the Caribbean rather than multiple independent introductions from the Old World.”, for “This pattern is consistent with a shared regional genetic background within the Caribbean/American group, although more molecular markers, more populations and specimens are needed to confirm this hypothesis.” (lines 182-184). Also, ”.. indicates that the species is present at multiple localities in northern Haiti. However, given the limited sampling effort, further surveys are needed to assess its wider distribution and establish the extent of its establishment.” (lines 185-188).

Also, we changed “…is compatible with a relatively recent arrival on Hispaniola. However, the available data do not allow us to determine the timing or pathway of introduction. Human-mediated transport may have contributed to its spread, but the relative importance of each introduction pathway remains unknown” (lines 190-193).

Importantly, we added the sentence: “Additional sampling across Haiti and the use of nuclear and/or genomic markers will be necessary to clarify the distribution, population structure and introduction pathways of the species.” (lines 197-199).

Also: “and may provide favorable conditions for the establishment” (line 202), and also: “This potential geographic suitability highlights the possibility of further spread into North America, Central and South America and underscores the value for strengthened surveillance and preparedness beyond the Caribbean.” (lines 215-217).

And, finally: “Our findings provide the first confirmed record of Aedes vittatus in Haiti and demonstrate its presence in multiple locations. Further surveillance is needed to determine its distribution, extent of establishment,…” (lines 218-220).

Inconsistencies in dates and sites / localities should also be corrected.

We have carefully checked and corrected inconsistencies in dates and sampling locations to ensure accuracy and coherence across the manuscript (e.g., sampling period until 31 October 2025 but identifications of Ae. vittatus between 7 and 24 of October). Also the number of localities and municipalities listed in Table I).

We have revised in the abstract “Larval surveys were done across 18 localities within 12 municipalities in the North and North-East departments of Haiti” (Lines 28-29).

In Results: “During the sampling period, Ae. vittatus was detected in 8 of 23 sites (34.7%) across..” (lines 137-138).

We added also more molecular identifications of other mosquito species, two species that are reported for the first time in Haiti.

In the abstract: “Additional mosquito species were identified, including first records of Cx. garciai and Cx. interrogator in Haiti.”

In results: “The COI sequencing confirmed the identification of Ps. pygmaea, Cx. quinquefasciatus, Ae. aegypti, Ae. albopictus and Cx. interrogator, with the latter showing high similarity to GenBank reference sequences (99.67% identity; MT999299.1). For Culex garciai, no reference sequences are currently available in GenBank; therefore, we generated the first COI sequences for this species in this study.“(lines 178-182).

We have also added some clarifications, regarding the molecular work. In material: “…as one of the five samples did not amplify,..” (line107).

In the discussion: “Notably, we also reported Cx. garciai and Cx. interrogator from Haiti for the first time. Morphological identification of Cx. interrogator was supported by molecular results, showing high sequence identity but lower query coverage, likely reflecting partial sequences or trimming. (lines 215-218).

References are generally relevant and updated, but their formatting should be standardized.

We have carefully reviewed and standardized the formatting of all references according to the journal guidelines. The reference list has been thoroughly checked for consistency in authors’ names, journal abbreviations, publication details, page ranges, and DOI formatting. All changes made to the references are indicated in red in the revised manuscript.

We believe these revisions have improved the clarity and rigor of the manuscript, and we thank you again for your helpful feedback.

Sincerely,

David Roiz, in behalf of the authors

# Reviewer report: REVIEWERS’ COMMENTS

## REVIEWER #1

No comments.
